# WAP four-disulfide core domain protein 2 promotes metastasis of human ovarian cancer by regulation of metastasis-associated genes

**DOI:** 10.1186/s13048-017-0329-0

**Published:** 2017-07-05

**Authors:** Yao Chen, Liping Huang, Suihai Wang, Tiancai Liu, Yingsong Wu, Ji-Liang Li, Ming Li

**Affiliations:** 1 School of Laboratory Medicine and Biotechnology, Southern Medical University, Guangzhou, 510515 China; 2 State Key Laboratory of Organ Failure, Guangdong Provincial Key Laboratory of Tropical Disease Research, Southern Medical University, Guangzhou, 510515 China; 3 Obstetrics and Gynecology Center, Nanfang Hospital, Guangzhou, 510515 China; 4 Institute of Antibody Engineering, Southern Medical University, 1023 Shatainan Road, Guangzhou, 510515 China; 5 Department of Oncology, Molecular Oncology Laboratories, Weatherall Institute of Molecular Medicine, University of Oxford, Oxford, OX3 9DS UK

**Keywords:** *WFCD2*, Ovarian cancer, Metastasis, Cell migration and invasion;

## Abstract

**Background:**

WAP four-disulfide core domain protein 2 (*WFDC2*) shows a tumor-restricted upregulated pattern of expression in ovarian cancer.

**Methods:**

In this study, we evaluated the role of *WFCD2* in tumor mobility, invasion and metastasis of ovarian cancer in clinical tissue and in ovarian cancer cells, both in vitro and in vivo.

**Results:**

Our results revealed *WFCD2* was overexpressed in ovarian tissues, and the expression level of *WFCD2* was associated with metastasis and lymph node metastasis. Higher expression of *WFCD2* was also observed in aggressive HO8910-PM cells than in HO8910 cells, and *WFCD2* knockdown halted cell migration, invasion, tumorigenicity and metastasis in ovarian cancer cells, both in vitro and in vivo. Knockdown of WFDC2 induced the down-regulation of ICAM-1, CD44, and MMP2.

**Conclusion:**

In summary, our work demonstrates that *WFCD2* promotes metastasis in ovarian cancer. These findings suggest that *WFCD2* plays a critical role in promoting metastasis and may constitute a potential therapeutic target of ovarian cancer.

**Electronic supplementary material:**

The online version of this article (doi:10.1186/s13048-017-0329-0) contains supplementary material, which is available to authorized users.

## Background

Among women, ovarian cancer is one of the most common gynecological cancers worldwide. With the highest mortality rate of all gynecologic cancers, ovarian cancer is very problematic to treat. Even after optimal treatment, more than half of patients suffer recurrence and eventually die [1].

Tumor progression is generally associated with extensive tissue remodeling to provide a proper environment for tumor growth, angiogenesis, invasion, and eventual metastasis of cancer cells. It is known that proteases are key agents in tumor progression, and that naturally expressed protease inhibitors have the ability to counteract tumor progression and metastasis [2–4]. However, expression of serine protease inhibitors (SPIs) in tumors is often associated with poor prognosis in cancer patients. Moreover, there is growing evidence that SPIs may even promote cancer cell malignancy which, if confirmed, could make them useful biomarkers of malignancy [5].

A recent study has identified *WFCD2* as a new member of the group of serine protease inhibitors belonging to the WAP family. While prior research indicated a direct linkage between *WFCD2* expression and cell proliferation [6, 7], its physiological and pathological mechanisms in tumorigenesis and metastasis have not been clearly elucidated.

Human *WFCD2* gene located on chromosome 20q12–13.1 locus, which encode a serial of proteins with a WAP-type four disulphide core (WFDC) domain [8, 9]. More and more evidence suggests that overexpression of WAP-type proteins closely related to tumor metastasis, especial *SLPI* and *P13* (encode antileukoproteinase 1 and elafin respectively). Both *SLPI* and *P13* are co-expressed with *WFCD2* and have been identified as a promoter in cancer development in various carcinomas [10, 11]. Expression of *SLPI* is positively correlated with increased expression of the cell cycle progression factor Cyclin D1 [12, 13], and its causal role in the promotion of malignant behavior has also been demonstrated in lung carcinoma cells stably transfected with human *SLPI*-expression constructs [14]. Elafin (*P13*) also has a role in counteracting environmental proteolytic conditions and repair-processes that are commonly associated with the inflammatory response, cancer progression, and invasion of various tumor cells [3, 15].

In view of the above information, the WAP proteins, had been considered as being associated with high-risk, metastatic, or aggressive cancer originating from various organs [9, 16]. We speculated that *WFCD2* might also play some role in tumor progression in ovarian cancer.

Our previous study indicated that knockdown of *WFCD2* induced the up-regulation of Fasl and down-regulation of Cyclin D1, as well as activating Caspase 3 and Ki67 [6]. These results indicate that *WFCD2* plays very important roles in tumor formation and proliferation. In the presented study, we analyze the expression of *WFCD2* in ovarian cancer cell line HO8910 and aggressively malignant line HO8910-PM. A cell model of *WFCD2* gene down-regulation was constructed and used to analyze the function of *WFCD2* in tumor metastasis and tumorigenesis in vitro and in vivo*.*

## Methods

### Ethic statement

Ovarian tumors were obtained from a cohort of patients treated at Nanfang Hospital, affiliated with Southern medical University, China, between 2011 and 2014. All research involving human ovarian cancer tissues have been approved by Nanfang hospital ethics committee and written consent was obtained from all participants. The 6- to 8-week-old female BALB/c–nu mice used in these experiments were provided by the experimental animal center of the Southern Medical University (Guangzhou, China). All mouse studies were approved by the Animal Ethics Committee of the Southern Medical University (Permit Number 20060015). All work was undertaken and that it conforms to the provisions of the declaration of Helsinki (as revised in Fortaleza, Brazil, October 2013).

### Patients and tissue samples

The median age of the patients was 50.8 years. All patients were diagnosed by pathological analyses based on the International Union Against Cancer (UICC) tumor node metastasis (TNM) stage system. 100 tissue samples (Table 1) from normal ovarian, primary tumors and matched adjacent non-neoplastic ovarian tissues were collected and prepared for Anti-*WFCD2* polyclonal antibody (Abcam, Cambridge, MA, USA) was used as primary antibody. The staining intensity (0, no staining; 1, weak staining; 2,moderate staining; and 3, intense staining) and the proportion of stained cells (0, no staining; 1, <10% staining; 2, between 11 and 33% staining; 3, between 34 and 66% staining; and 4, >67% staining) were semiquantitatively determined. The intensity and the percentage of positive cell scores were multiplied (0–12) and classified into three groups: weak (0–4), moderate (5–8) and strong (9–12). All slides were scored by two observers blinded to the pathology and the clinical features.

**Table 1:** Distribution by tumor characteristics for ovarian cancer patients

Variable	No. patients (%)	
	n	%
Total		
Age(years)		
≤ 50	38	38
> 50	62	62
FIGO stage		
Stage I	26	28.57
Stage II	21	23.08
Stage III	31	34.07
Stage IV	12	13.19
Grade(Epithelial, *n* = 91)		
G1	29	31.87
G2	46	50.55
G3	16	17.58
Histological type		
Serous cystadenocarcinoma	46	50.55
Mucinous cystadenocarcinoma	22	24.18
Endometrioid tumor	14	15.38
Clear cell cacinoma	9	9.89
Transcoelomic Metastasis		
No	65	71.42
Yes	26	28.57
Lymph node metastasis		
No	74	81.31
Yes	17	18.69

### Cell lines and reagents

Human ovarian cancer cell lines SKOV3, HO8910 and HO8910-PM were purchased from the cell bank of the Chinese Academy of Sciences (Shanghai, China). The cells were maintained in Dulbecco’s modified Eagle’s medium (DMEM) media supplemented with 10% fetal calf serum (FBS) in an atmosphere of 5% CO2 at 37 °C. Restriction enzymes from TAKARA (TaKaRa Bio, Inc., Shiga, Japan); Transwell system from CostarCorning (Corning, NY, USA).; Puromycin and Trizol reagent from Invitrogen (Life Technologies, Carlsbad, CA, USA); cell culture media (antibiotic, serum and glutamine) from GIBCO (Life Technologies, Carlsbad, CA, USA). All other molecular reagents and solvents were purchased from SIGMA Corp (St. Louis, MO, USA).

### Gene knockdown

*WFCD2* knockdown was conducted in low-passage (<20) ovarian cancer cells. The shRNA oligo sequences were designed to against the human *WFCD2* gene (Gene Bank Accession No. NM_0006103.3). The shRNA sequence against *WFCD2*(5′-GCTCTCTGCCCAATGATAAGG-3′) and a invalid RNAi sequence(5′-GTTCTCCGAACGTGTCACGT-3′) were chemically synthesized and constructed into the lentiviral by Shanghai Genepharma Co.Ltd. The *WFCD2*-specific shRNA lentivirus paticals was collected and transfected into the HO8910 and SKOV3 cell lines. For stable knockdown of *WFCD2*, the transfected HO8910 and SKOV3 cell lines, named HO8910–209 and SKOV3–209 respectively, were selected by Puromycin. Puromycin-resistant colonies were picked and expanded separately.

### RNA extraction and real-time RT-PCR

Total RNA was isolated with Trizol regents and reverse transcription was performed using the PrimeScript 1st Strand cDNA Synthesis Kit, according to the manufacturer’s instructions, cDNA samples (0.1 μg) were assayed in duplicate using the ABI Prism 7500 detection system (Life Technologies, Carlsbad, CA, USA). Using the SYBR Green PCR Master Mix (TaKaRa) following protocols. The relative quantization number was then calculated by subtracting the average CT from the corresponding average CT for β-actin.

### Tumour migration assay

Transwell polycarbonate plates with 6.5 mm diameter tissue culture inserts containing a membrane with 8 μm pores were used for migration assay. Low passage (<20) cells were cultured in the medium without serum to synchronize most of them at G1/G0 and then suspended in serum-free DMEM and seeded (5 × 10^4^ cells/well) into each insert. The condition medium with 10% FBS collected after 24 h culture was added to each outer well. The plates were then assembled and incubated for 8 h at 37 °C, 5%CO2. After a 8 h incubation, the plates were rinsed once in PBS, fixed in 70% alcohol for 10 min, and rinsed with 0.5% crystal violet. Cells adhering to the top surface of the tissue culture inserts were removed with a cotton tip applicator, while cells adhering to the bottom surface of the inserts were rinsed with 1% Triton-X 100 in PBS for 20 min. The membranes of the tissue culture inserts were viewed under amicroscope (10× magnification) and the number of cells in 4 random fields was determined.

### Tumour invasion assay

For invasion assays, 5 × 10^4^ cells (cell passage <10) were cultured in the medium without serum to synchronize most of them at G1/G0 and then plated in DMEM/1% FBS in a cell invasion chamber (Transwell Cell invasion assay kit, Corning) in a 24-well plate, which contained an 8 μm pore size polycarbonate membrane covered with a thin layer of collagen matrix. Invasive cells migrated through a membrane according to the gradient of FBS to the lower chamber, which contained DMEM/15% FBS. The invasive cells were stained with crystal violet, and the number of cells in 4 random 10× magnification fields was determined.

### In vivo tumor formation and peritoneal dissemination

For the generation of intraperitoneal tumors, HO8910-NA cells and HO8910–209 cells were injected intraperitoneally (i.p.) into mice (*n* = 10). Each mouse received one injection of 3 × 10^6^cells. Animals were monitored 3 times weekly for tumor formation. All injection-treated mice were fed for 10 weeks after injection. At the end of 10 weeks, all the mice were sacrificed and the abdominal region examined for tumor formation. Each tumor burden in the peritoneal cavity was weighed and collected and paraffin-preserved according to the usual protocols.

### Western blot

Total protein was extracted by sonication in radio-immunoprecipitation assay (RIPA) buffer(50 mM Tris–HCl pH 7.5, 150 mM NaCl, 5 mM EDTA, 0.5% Nonidet P-40, 5 mM dithiothreitol, 10 mM NaF, protease inhibitor cocktail). 100 μg denaturedprotein was separated on an SDS-polyacrylamide gel and transferred to Hybond membrane (Amersham, Germany), which was then blocked overnight in 5% skimmed milk inTris-bufferedsaline with Tween 20 (TTBS, 10 mM Tris–HCl, 150 mM NaCl, 0.1% Tween 20). For immunoblotting, the membrane was incubated for 15 min with antibodies. The membrane was rinsed with TBST and incubated with anti-mouse, anti-rabbit or anti-goat IgG conjugated to horseradish peroxidase (DAKO, USA, 1:1000) for 15 min. All the incubations were performed in a microwave oven to allow intermittent irradiation. Bands were visualized with LAS4010 (GE Healthcare Life Science, USA) by ECL-Plus detection reagents (Santa Cruz, USA). Densitometric quantification of protein bands was performed with GAPDH as an internal control using Image J (NIH, USA).

### Immunohistochemistry

Immunohistochemistry was done using a single-staining procedure. Anti-WFCD2, anti-Ecadherin,anti-Vimentin monoclonal antibody (Cell Signaling Technology), anti-CD44,anti-MMP2,anti-MMP9,and anti-ICAM-1 rabbit polyclonal antibody (Santa Cruz Biotechnology), were applied to the slides at a dilution of 1:1,00 ~ 1:150 in blocking buffer overnight at 4 °C. The slides were then washed and stained by the avidin-biotin method. The slides were lightly counter stained with hematoxylin. Tumor cells were considered positive for the antigen if there was brown color staining. The intensity was scored as negative (0), weak (1), medium (2), and strong (3),and the proportion of staining was scored as 1 (≤10%), 2 (11–50%), 3 (51–75%), and 4 (>75%). An overall expression score was calculated by multiplying the scores for intensity and proportion, ranging from 0 to 12. For ICAM-1, at least 500 tumor cell for each xenograft sample (*n* = 5) were randomly selected and counted. The number of positive cell was counted and the positive index was calculated as follows: ICAM-1 index = (number of stained cells/total cell number) × 100%.

### Statistical analysis

All experiments were performed at least in triplicate. All data are reported as the mean ± standard deviation. Using Excel 2007 (Microsoft Corporation, Redmond, WA, USA). Microsoft Office Excel 2007 (Microsoft Corporation, Redmond, WA, USA) and the statistical software SPSS13.0 (SPSS Inc., Chicago, IL, USA) were used in data processing and analyzing the significance with the one-way ANOVA,t-Test,or the log rank test (for Kaplan-Meier plots). Results with *P* < 0.05 were considered statistically significant.

## Results

### Increased expression of *WFCD2* correlated with the progression and peritoneal metastasis of human ovarian cancer

**Fig. 1:**
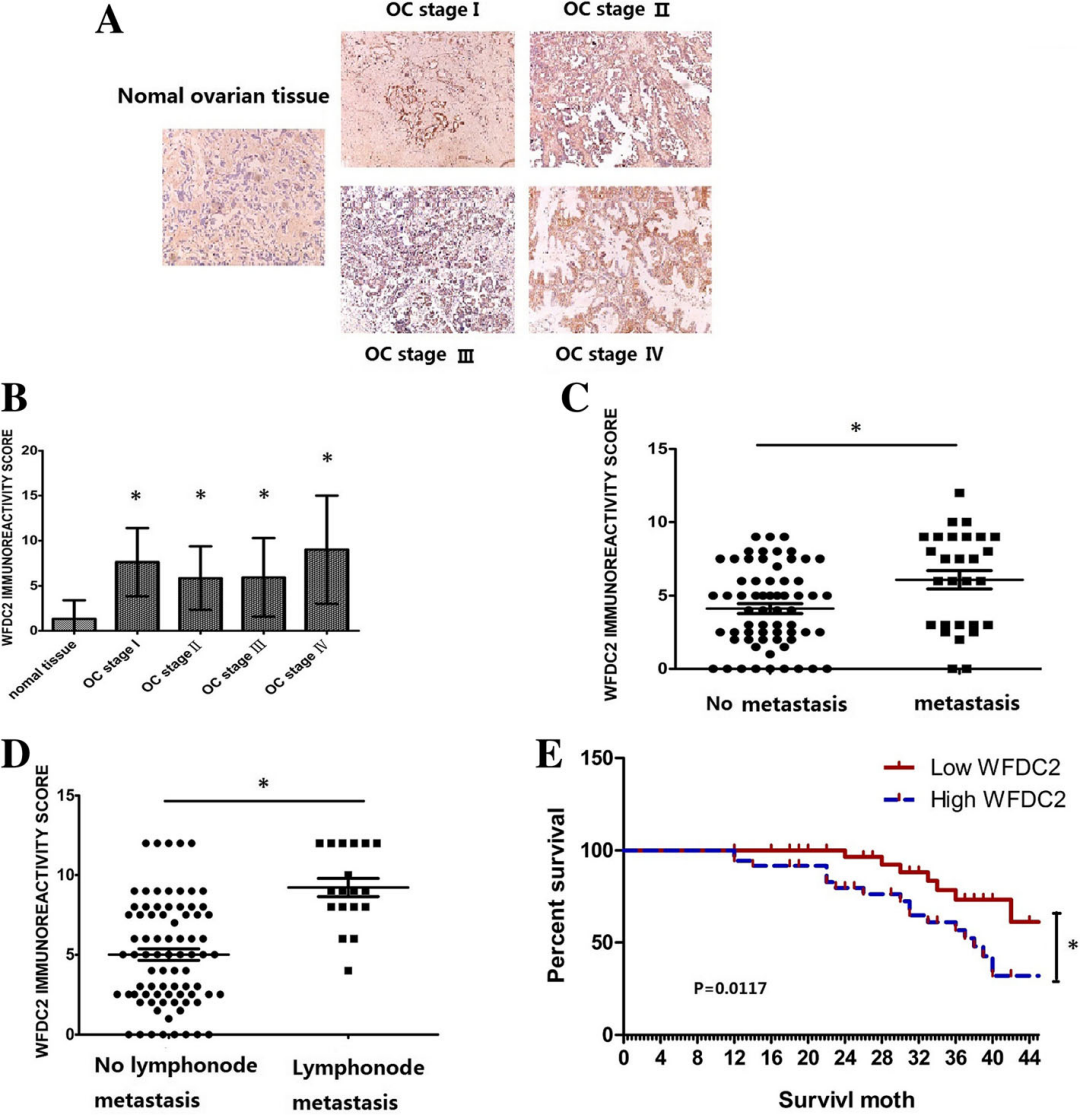
Over-expression of *WFDC2* was correlated with stage and peritoneal metastasis in ovarian cancer. **a** Representative images of *WFDC2* expression in normal ovarian tissue and primary ovarian cancer tissues (FIGO I, II, III, and IV) are shown (200× magnificaiton); **b** *WFDC2* is significantly increased in specimens from ovarian cancer tissue compare with the normal ovarian tissue(* *P* < 0.05;); **c** *WFDC2* scores are significantly higher in primaray tumors with metastasis than in non-metastasis specimens(* *P* < 0.05;). **d** *WFDC2* scores are significantly higher in primaray tumors with lymph mode metastasis than in non-lymph mode metastasis specimens(* *P* < 0.05;).(**E**) Kaplan-Meier survival curves for 72 ovarian cancer patients, stratified based on individual *WFDC2* expression(* *P* < 0.05;)

To examine the potential clinical relevance of *WFCD2* to ovarian cancer progression, the human ovarian cancer tissues were derived from patients with progressive ovarian disease to investigate *WFCD2* expression and its association with different clinicopathological parameters. Undetectable to very low *WFCD2* staining were observed in normal ovarian tissue, whereas ovarian carcinomas showed higher *WFCD2* immunoreactivity in most cases (Fig. 1a). The *WFCD2* staining score of carcinomas is significantly higher than that of non-neoplastic ovarian tissues (all FIGO stage), which revealed a correlation between *WFCD2* expression levels and ovarian cancer progression (Fig. 1b), while no significence had been observed between high-grade carcinomas and low-grade carcinomas. Next, the correlation between *WFCD2* expression and key clinical parameters in human ovarian cancer were assessed. There were no significant correlations between high *WFCD2* expression and patient age or histological subtypes. While consistent with the results in migration and invasion assay in vitro, peritoneal transcoelomic dissemination was positively associated with the expression of *WFCD2*. The expresoion of *WFCD2* was higher in primary tumors with peritoneal metastasis and lymph node metastasis sample (Fig. 1c-d). The Kaplan-Meir survival graphs in Fig. 1e display the association of WFDC2 expression with ovarian cancer survival. As shown in Fig. 1e, the degree of expression of WFDC2 were significantly associated with longer survival in EC (log rank *p* = 0.0117).Taken together, these data strongly indicate that enhanced *WFCD2* may play a role in the progression of the primary ovarian cancer cell to peritoneal metastasis.

### Expression of *WFCD2* mRNA and protein in HO8910 and HO8910-PM cells

**Fig. 2:**
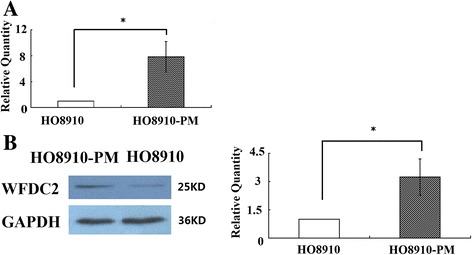
Expression of *WFDC2* in HO8910 cell line and high metastatic potential HO8910PM cells. **a** Normalized *WFDC2* genes mRNA levels in the HO8910 and HO8910-PM. The relative quantities of *WFDC2* protein were determined by densitometry and normalized by using β-actin. *P < 0.05 compared to HO8910; **b** Western blot analysis of expression of *WFDC2* and *GAPDH.* Normalized *WFDC2* protein levels in ovarian cancer cell lines HO8910 and HO8910PM. The relative quantities of *WFDC2* protein were determined by densitometry and normalized by using *GAPDH.* *P < 0.05 HO8910-PM compared to HO8910

HO8910 is a poorly differentiated human ovarian serous adenocarcinoma cell line and removed from a patient with serous ovarian Cystadeno carcinoma as well as ascites. The HO8910PM cell line was derived from HO8910 grown as xenografts in nude mice and was considered to possess more invasive and metastatic potential than HO8910 cell lines [17]. Real-time RT-PCR showed that *WFCD2* mRNA was expressed more strongly in HO8910PM cells, at levels 8 times higher than that observed in the HO8910 cells (*p* < 0.005) (Fig. 2a). Secreted *WFCD2* protein was detected only at very low levels in the medium containing HO8910 cells, but was amplified three folds in the HO8910-PM cells by comparison (*p* < 0.005) (Fig. 2b). These results, with significantly different levels of *WFCD2* expression between HO8910 and HO8910PM, indicate that *WFCD2* might be involved in cell invasion and the metastasis of ovarian cancer cells.

### *WFCD2* knockdown reduces cell migration, invasion

**Fig. 3:**
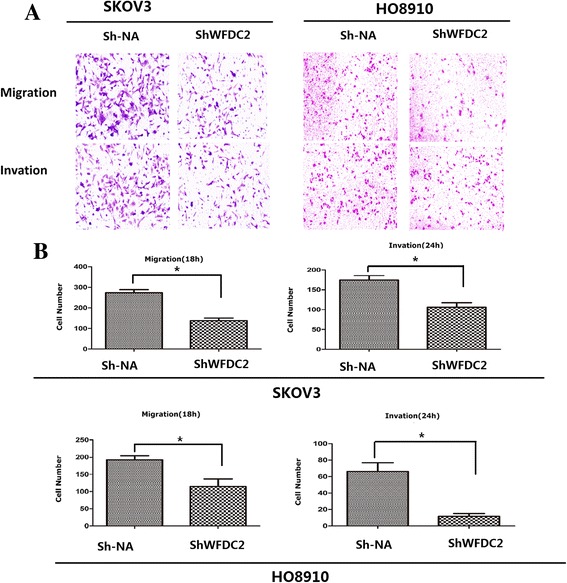
*WFCD2* knockdown reduces cell migration, invasion. **a** Assessment of cell migration and invasion of *WFDC2* knockdown lines and control. **b** Quantification of the migration and invasion is expressed as the number of invasive cells per HPF. Bottom, representative micrographs of the transwell migration and invasion assay (10× magnification) * *P* < 0.05 ** <0.01 *WFDC2* knockdown (sh-*WFDC2*) compared to negative control (sh-NA)

*WFCD2* knockdown was conducted in HO8910 and SKOV3 ovarian cancer cells (see Additional file 1: Figure S1). Before we did the cell migration and invasion assay, we had considered that the knockdown of WFDC2 cause slower growth and reduced cycling D level and may cause confused in the result. So before we seed the cells into the culture insert, we starved the cell for 12 h by culturing in the medium without serum. So all the cells would be synchronize most of them at G1/G0 to reduce the confounding effects of reduced cell proliferation in the knockdown cells. The transwell system was used for migration and invasion assay. The migration assay showed that the number of *WFCD2* knockdown cells passing through the membrane was much lower than the control cells (Fig. 3a, b). In the invasion assay, a polycarbonate membrane over which a thin layer of extracellular matrix (ECM) was applied served as an in vitro basement membrane. Since only invasive cells are capable of migrating through the ECM layer, we observed that the number of cells passing through the ECM layer was much lower in the *WFCD2* knockdown group than the control. This result indicated that cells of control group readily passed through the membrane, the *WFCD2*-knockdown cells were in large part unable to invade the matrix, (Fig. 3a, b), indicating that overexpression of *WFCD2* in ovarian cancer might heighten the potential for tumour metastasis.

### *WFCD2* knockdown reduces and peritoneal implantation potential of ovarian cancer cells

**Fig. 4:**
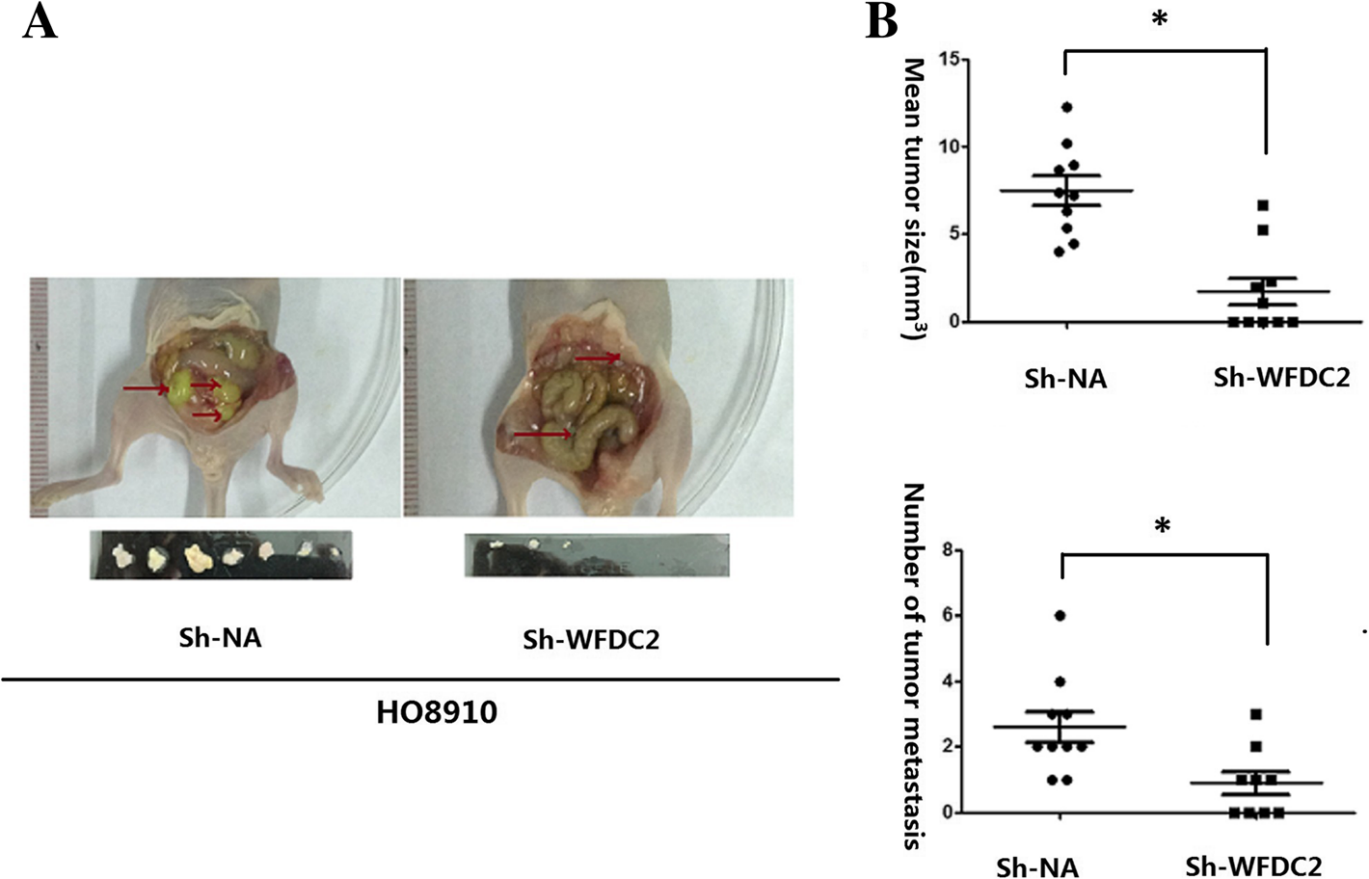
*WFDC2* Promoted the metastasis and of peritoneal implantation of ovarian cancer cell. **a** *WFDC2* knockdown suppressed growth of HO8910 ovarian cancer cells in the peritoneum compared with the negative control on week 10 after transplanting. **b** Effect of *WFDC2* knockdown on peritoneal dissemination was assessed by counting the number of nodules and evaluating tumor size in the mesentery and peritoneal wall compared with controls. Ten mice were used in each group. Each value represents mean ± s.e. * *P* < 0.05, *WFDC2* knockdown (sh-*WFDC2*) compared to negative control (sh-NA)

Tumor growth and peritoneal dissemination was also evaluated in vivo. We utilized the intraperitoneal xenograft mouse model with *WFCD2* knockdown and the control cells. 10 weeks after tumor inoculation, mice were sacrificed and we measured the peritoneal dissemination of the mice. Figure 4a shows the effects of *WFCD2* knockdown on the peritoneal dissemination of ovarian cancer cells. In the negtive control group, many peritoneally disseminated tumors (marked with arrows) were observed, whereas in the *WFDC2* knockdown mice peritoneal dissemination was clearly suppressed. Peritoneal disseminated tumors had been observed in 8 of 10 mice in the negtive control group group, while only 3 mice in *WFDC2* knockdown group were found to have tumor metastases. Peritoneal and mesenteric nodules were observed in the liver (6/10), intestines (8/10) and abdomen (3/10) in the mice of the negtive control group. However, in mice of the *WFDC2* knockdown group, peritoneal and mesenteric nodules were absent from abdominal organs, though some were found in the intestine (3/10). These data indicate that, compared with the control group, the growth and metastasis of tumors were markedly inhibited by *WFCD2* gene knockdown (Fig. 4b). The sizes and number of peritoneal and mesenteric nodules at 10 weeks after the transplantation of *WFDC2* knockdown cells were significantly smaller and fewer than that of the control group (Fig. 4b).

### The pro-malignant activity of *WFCD2* is mediated by its effect on *CD44,MMP2* and *ICAM-1* expression

**Fig. 5:**
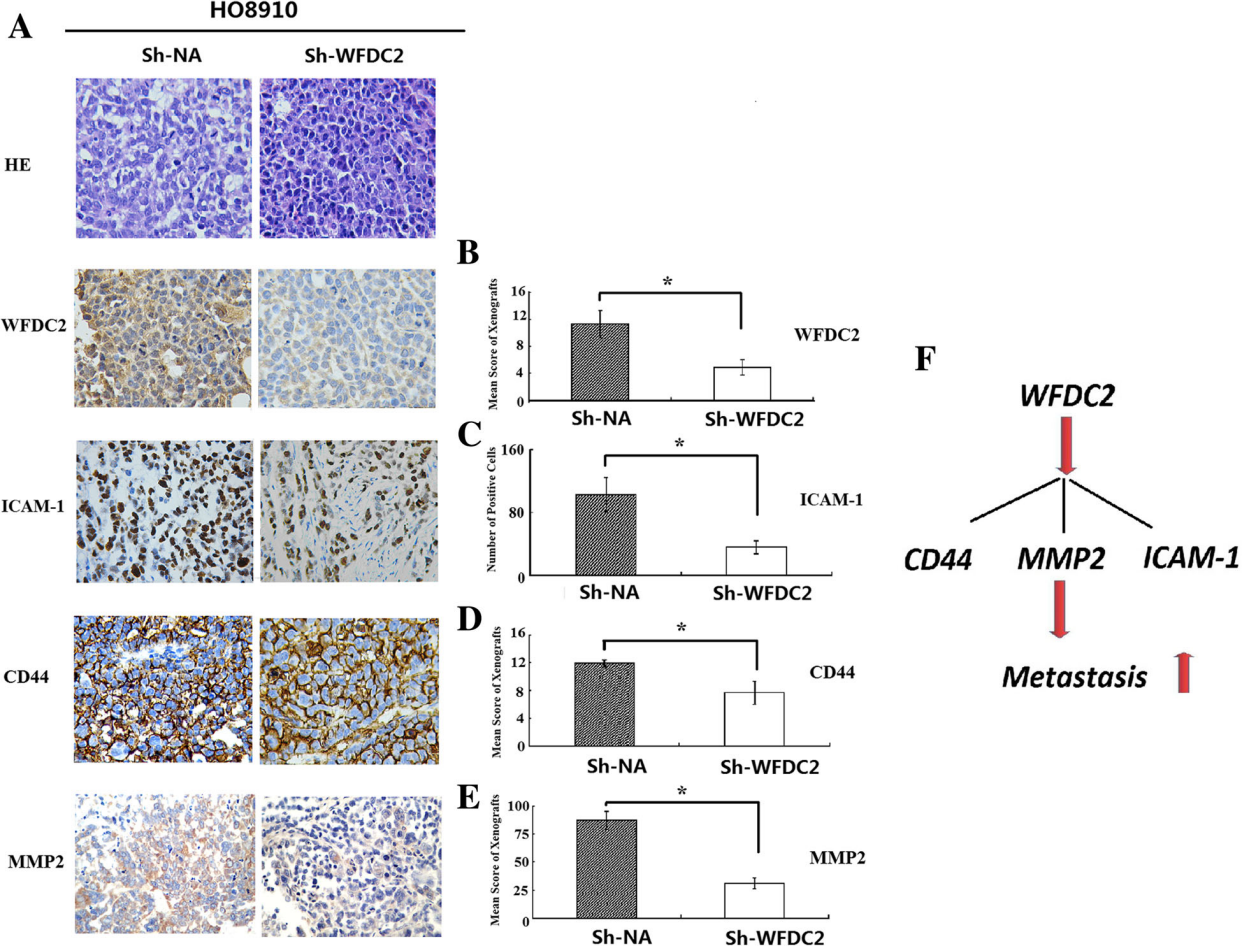
The expression of ICAM-1,MMP2 and CD44 on *WFDC2* and *WFDC2* knockdown cells. **a** *WFDC2*,ICAM-1 and CD44 immunohistochemistry in WFDC2 knockdown and negative control xenografts; Xenograft tumor sections (HO8910) were stained with either anti-WFDC2 antibody,anti-CD44 antibody,anti-MMP2 or anti-ICAM-1 antibody. **b, c, d, e**.Normalized *WFDC2*,ICAM-1,MMP2and CD44 protein levels in *WFDC2* knockdown and negtive control xenografts. *P < 0.05 *WFDC2* knockdown compared to negative control; **f** Summary of *WFDC2*-mediated molecular effects on ovarian cancer metastasis

Since reduced exogenous secretion of *WFCD2* in *WFCD2* gene knockdown mice resulted in a significant suppression of tumorigenesis and metastasis. We suspect that *WFCD2* promotes the peritoneal implantation of ovarian cancer cells. To explore the mechanisms of tumor inhibition by *WFCD2* knockdown in vivo, the animals were sacrificed and the expression of the genes related to tumorigenesis and metastasis were detected by immunohistochemistry (CD44,ICAM-1,VCAM-1,MMP2,MMP9). In our study, CD44 and MMP2 staining showed that membrane localization of this protein was reduced when *WFCD2* was depleted (Fig. 5a, d, f). We also found that the number of positive cells in the ICAM-1 staining was lower in the *WFCD2* knockdown cells compared to the control (Fig. 5a, c). The IHC results had been comfirmed by qRT-PCR (Additional file 1: Figure S2). These results confirm the relationship between the *WFCD2* and cell tumorigenesis and metastasis of cancer cells.

## Discussion

Various regulators are involved in the processes of malignancy and metastasis. Recent evidence suggests that proteins of the WAP family play an important role in tumor progression, malignancy, and metastasis [9, 18].

*WFCD2* is one of the members of the WAP family and previous research has shown that the blood concentration of *WFCD2* is higher in patients with ovarian cancer than in women with either healthy ovaries or benign ovarian tumors [8, 19, 20]. Thus we hoped to identify the role of *WFCD2* in the malignancy and metastasis of ovarian cancer. In the present study, we analyzed the expression of *WFCD2* in ovarian cancer cell lines HO8910 and HO8910PM. The latter is derived from HO8910 and is considered to have more potency in invasion and metastasis than its parent HO8910 cell lines. We observed a higher expression of *WFCD2* in both the RNA and protein level in HO8910PM cells than that in HO8910 cells. These data indicate that *WFCD2* may be a tumor-specific gene involved in the malignancy and metastasis of ovarian cancer. A comparable correlation between *WFCD2* expression and the malignant behavior of ovarian carcinoma cells has also been established.

Our results shows that the high expression of *WFCD2* is ovarian cancer tissues of all FIGO stages, and positively correlated to lymph node metastasis (*p* < 0.05) and implanted metastasis (*p* < 0.05), which indicates that high expression of *WFCD2* may relate to the progression of ovarian cancer. Hence, *WFCD2* may be a potential biomarker of clinical staging and may possibly be a biomarker for prognosis assessment. However, the application value of clinical examination still needs further evaluation.

After knockdown of *WFCD2* expression, the invasion and migration rate was significantly lower in the *WFCD2* cells compared to the blank control both in HO8910 and SKOV3 cells. The decreased malignancy of these ovarian cancer cells was confirmed to be associated with the lower levels of *WFCD2*. This suggests that *WFCD2* accelerates the migration and invasion of ovarian cancer cells, as expected.

Unlike most solid tumors, ovarian cancer spreads mainly via implantation within the peritoneal cavity, and hematogenous metastasis is seldom observed [18, 21–23]. The invasive and migratory capacity of ovarian cancer cells plays a key role in the metastasis process. To determine if the changes observed in vitro as a result of *WFCD2* knockdown are reproducible in vivo, we established an ovarian cancer xenograft model. This in vivo study using *WFCD2* gene-knockdown ovarian cancer cell HO8910–209 showed that *WFCD2* knockdown suppressed both ovarian tumor growth and peritoneal dissemination. The type of destination organs, the number of metastases, and the amount of nodules in HO8910–209 groups were significantly lower than in the control groups. These results are consistent with the results in vitro, and confirm that *WFCD2* knockdown inhibits cell migration and invasion, thereby inhibiting the malignancy and metastasis of ovarian cancer.

Many factors can affect the tumour metastasis. Our previous study indicated that knockdown of *WFCD2* induced cell apoptosis and depressed cell proliferation [6]. In this study, several biochemical markers used to characterize metastases had been evaluated by immunohistochemical methods to further elucidate the role of *WFCD2* in tumorigenicity in vivo. In these biochemical markers, such as ICAM-1, VCAM-1, CD44, MMP2, MMP9, we observed that the expression of CD44, MMP2 and ICAM-1 was significantly reduced in *WFCD2* knockout tumor cells, which might explain WFCD2 knockdown reduced the mobility of tumor cells both in vitro and in vivo (Results are schematically summarized in Fig. 5f). ICAM-1 is an important cell-adhesion molecule directly linked to ovarian tumor growth, metastasis and chemo-resistance [24]. CD44 is a receptor for hyaluronic acid,up-regulation of CD44 represents a crucial event in the development of metastasis, recurrence, and drug resistance to current treatments in ovarian cancer. MMP-2 (along with MMP-9) is capable of degrading type IV collagen, the most abundant component of the basement membrane. The interaction of MMP2 and CD44 is an important factor in selectively regulating the tumor microenvironment to promote tumor cell metastasis and is considered to be an inducer of EMT [22, 25, 26]. To be interesting, Hokins etl had also reported that paracrine SLPI secretion upregulated MMP2 and MMP9 transcription and secretion in some cancer cells [24, 25]. All this suggests a role for *WFCD2* in rebuilding the tumor microenvironment by regulating the expression of MMP2 and CD44. As both CD44 and MMP2 are inducer of epithelial-mesenchymal-transition (EMT),which strengthens our confidence that *WFCD2* might participate in tumor metastasis and disease processes by regulating the progression of EMT in ovarian cancer cells. However, the role of *WFCD2* as a regulator in EMT is still required further evaluation.

## Conclusion

In summary, we show that *WFCD2*, which is up-regulated in ovarian cancer, can now for the first time be considered a regulator of tumor metastasis. These results provide fresh impetus for further exploration into metastasis in ovarian cancer. We propose *WFCD2* as a potential therapeutic target and prognostic marker for ovarian cancer and believe that uncovering these processes will provide key information that extends our understanding of the occurrence and development of ovarian cancer and its treatment.

## Additional file

Additional file 1: Figure S1. Expression of WFDC2 in WFDC2 knockdown Clonal Lines. (A) Western blot analysis of expression of *WFDC2* and *GAPDH* in SKOV3 cells*.* Normalized *WFDC2* protein levels in the *shRNA*-transfectant, mock-transfectant NA and control. The relative quantities of *WFDC2* protein were determined by densitometry and normalized by using *GAPDH.* **P* < 0.05 compared to mock-transfectant sh-NA; #*P* < 0.05 compared to SKOV3. (B) Western blot analysis of expression of *WFDC2* and *GAPDH* in HO8910 cells*.* Normalized *WFDC2* protein levels in the *shRNA*-transfectant, mock-transfectant sh-NA. The relative quantities of *WFDC2* protein were determined by densitometry and normalized by using *GAPDH.* **P* < 0.05 compared to sh-NA; **Figure S2.** The genes related to metastasis were modified by *WFDC2* knockdown. Normalized metastasis related genes mRNA levels in the *WFDC2* knockdown and negative control cells. The relative quantities of *WFDC2*,ICAM-1,CD44 and MMP2 mRNA were determined by densitometry and normalized by using β-actin. **P* < 0.05 compared to sh-NA. (DOC 470 kb)

## Abbreviations

CD44: Cluster of Differentiation 44
EMT: Epithelial mesenchymal transition
ICAM-1: Intercellular adhesion molecule
IHC: Immunohistochemistry
MMP2: Matrix metalloproteinase-2
MMP9: Matrix metalloproteinase-9
NC: Negative control
qPCR: Quantitative real-time PCR
VCAM-1: Vascular cell adhesion molecule 1
*WFDC2*: WAP four-disulfide core domain 2
