# Supplementary material for: WAP four-disulfide core domain protein 2 promotes metastasis of human ovarian cancer by regulation of metastasis-associated genes
Source: J Ovarian Res. 2017 Jul 5;10:40. doi: 10.1186/s13048-017-0329-0 (PMC5499000; doi:10.1186/s13048-017-0329-0)
Supplement: Additional file 1: Figure S1. — Expression of WFDC2 in WFDC2 knockdown Clonal Lines. (A) Western blot analysis of expression of WFDC2 and GAPDH in SKOV3 cells. Normalized WFDC2 protein levels in the shRNA-transfectant, mock-transfectant NA and control. The relative quantities of WFDC2 protein were determined by densitometry and normalized by using GAPDH. *P < 0.05 compared to mock-transfectant sh-NA; #P < 0.05 compared to SKOV3. (B) Western blot analysis of expression of WFDC2 and GAPDH in HO8910 cells. Normalized WFDC2 protein levels in the shRNA-transfectant, mock-transfectant sh-NA. The relative quantities of WFDC2 protein were determined by densitometry and normalized by using GAPDH. *P < 0.05 compared to sh-NA; Figure S2. The genes related to metastasis were modified by WFDC2 knockdown. Normalized metastasis related genes mRNA levels in the WFDC2 knockdown and negative control cells. The relative quantities of WFDC2,ICAM-1,CD44 and MMP2 mRNA were determined by densitometry and normalized by using β-actin. *P < 0.05 compared to sh-NA. (DOC 470 kb) [file 13048_2017_329_MOESM1_ESM.doc]

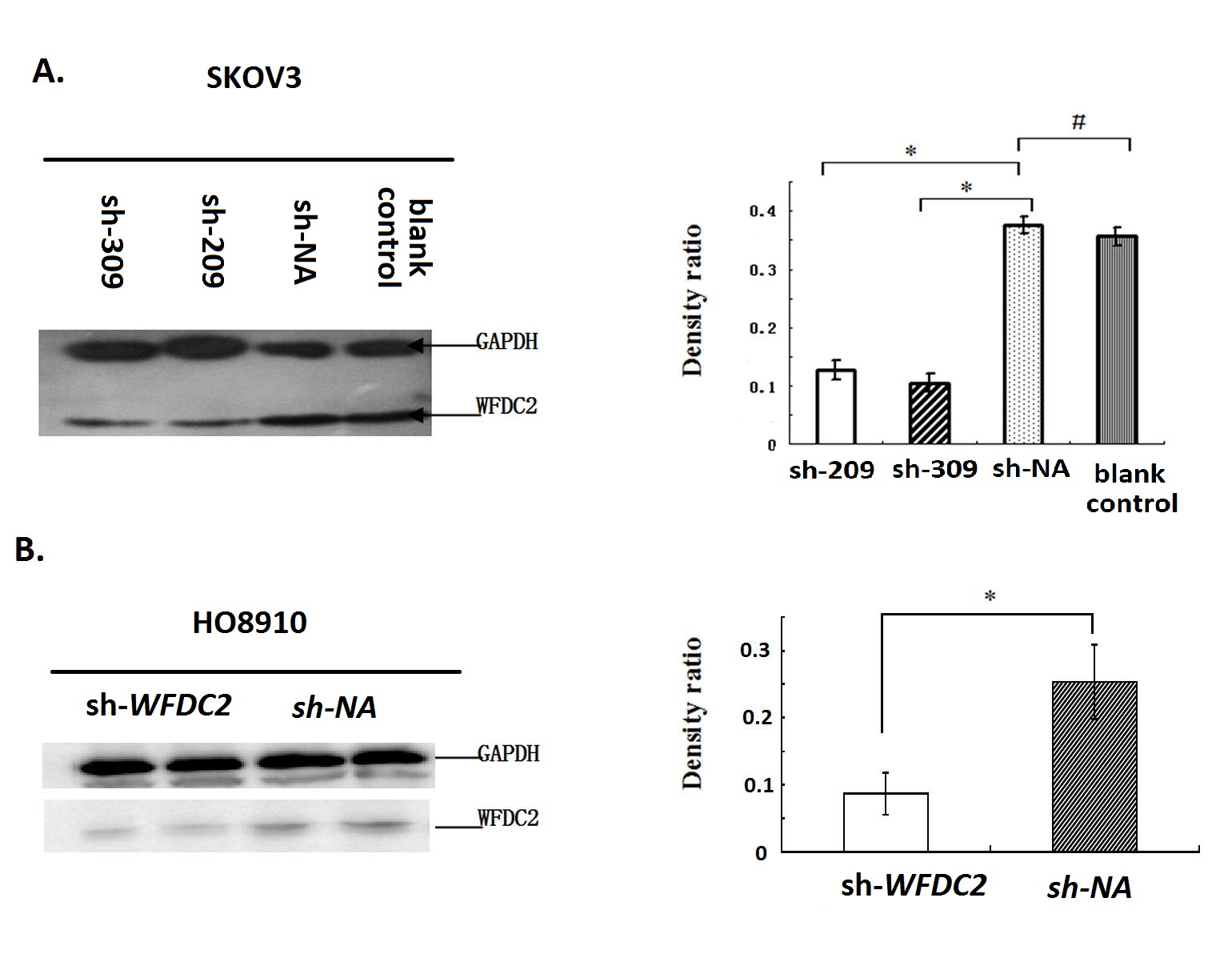


**Figure. S1** **Expression of WFDC2** **in WFDC2 silences Clonal Lines.**

**（A）**Western blot analysis of expression of *WFDC2* and *GAPDH* in SKOV3 cells*.* Normalized *WFDC2* protein levels in the *shRNA*-transfectant, mock-transfectant NA and control. The relative quantities of *WFDC2* protein were determined by densitometry and normalized by using *GAPDH.* *P <0.05 compared to sh-NA; #P <0.05 compared to mock control. **（B）**Western blot analysis of expression of *WFDC2* and *GAPDH* in HO8910 cells*.* Normalized *WFDC2* protein levels in the *shRNA*-transfectant, mock-transfectant NA. The relative quantities of *WFDC2* protein were determined by densitometry and normalized by using *GAPDH.* *P <0.05 compared to sh-NA;

**
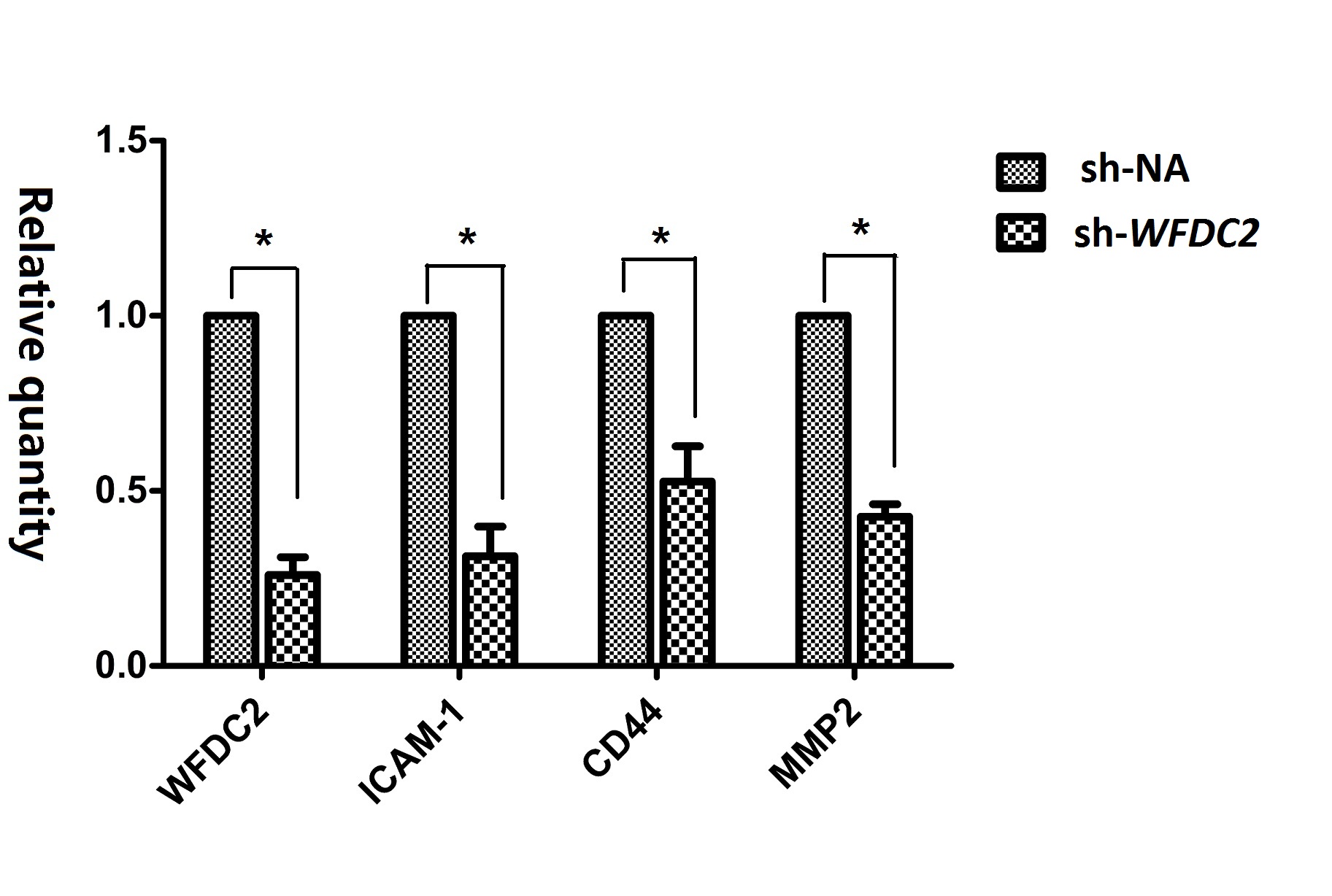
**

**Figure.S2 The genes related to metastasis were modified by *WFDC2* knockdown**

Normalized metastasis related genes mRNA levels in the *WFDC2* knockdown and negtive control cells. The relative quantities of *WFDC2*,ICAM-1,CD44 and MMP2 mRNA were determined by densitometry and normalized by using β-actin. *P <0.05 compared to sh-NA;
